# PTPRO promoter methylation is predictive of poorer outcome for HER2-positive breast cancer: indication for personalized therapy

**DOI:** 10.1186/1479-5876-11-245

**Published:** 2013-10-03

**Authors:** Yi-Teng Huang, Fei-Fei Li, Chen Ke, Zhou Li, Zong-Tai Li, Xiao-Fang Zou, Xiao-Xuan Zheng, Yu-Ping Chen, Hao Zhang

**Affiliations:** 1 Department of Integrative Oncology, Affiliated Cancer Hospital of Shantou University Medical College, Shantou 515041, China; 2 Tumor Tissue Bank, Affiliated Cancer Hospital of Shantou University Medical College, Shantou 515041, China; 3 Cancer Research Center, Shantou University Medical College, Shantou 515041, China; 4 Department of Thoracic Surgery, Affiliated Cancer Hospital of Shantou University Medical College, Shantou 515041, China

**Keywords:** Breast cancer, PTPRO, Methylation, HER2, Prognosis

## Abstract

**Background:**

Protein Tyrosine Phosphatase Receptor-type O (PTPRO) has recently been in the spotlight as a tumor suppressor, whose encoding gene is frequently methylated in cancers. We examined the methylation status of the PTPRO gene promoter in breast cancer and evaluated the correlation between PTPRO promoter methylation and both clinicopathological parameters and prognosis of breast cancer patients.

**Methods:**

Two hundred twenty-one formalin-fixed, paraffin-embedded (FFPE) tumor tissues, 20 FFPE normal adjacent tissues and 24 matched plasma samples, collected from primary breast cancer patients, were assessed for PTPRO gene promoter methylation using methylation-specific PCR. Associations of promoter methylation with clinicopathological parameters were evaluated. Kaplan-Meier survival analysis and Cox proportional hazards models were used to estimate the effect on survival.

**Results:**

175 samples gave identifiable PCR products, of which 130 cases (74.3%) had PTPRO gene promoter methylation. PTPRO methylation correlated with higher histological grade (*P* = 0.028), but not other clinical parameters. Multivariate analysis indicated that overall survival (OS) was significantly poorer in HER2-positive, but not ER-positive patients with methylated-PTPRO. Methylated-PTPRO was detectable in matched plasma samples and only observed in plasma from patients whose corresponding primary tumors were also methylated.

**Conclusions:**

PTPRO methylation is a common event in the primary breast cancer and can be reliably detected in peripheral blood samples. PTPRO methylation is associated with poor survival only in HER2-positive patients, suggesting use of PTPRO methylation as a prognostic factor for breast cancer and for optimizing individualized therapy for HER2-positive patients.

## Background

As the most common malignancy and the second leading cause of cancer related deaths in women [1], breast cancer is a heterogeneous disease with a variety of pathological entities and varied clinical behavior [2]. The past decade has brought together substantial advances in comprehensive molecular profiling and a maturation of understanding in tumor biology [3,4]. Such studies provide a more precise molecular stratification of patients and allow us to better understand the clinical behavior and the targets for better therapy [5-7]. Despite these improvements, numerous problems remain. For example, endocrine therapies and newly developed targeted drugs (Trastuzumab and Lapatinib) provide longer survival, but a number of patients encounter either de novo or acquired resistance [8-10]. Furthermore, a fraction of patients suffer severe adverse events, such as thrombosis and cerebrovascular or cardiac deaths [11]. Studies on molecular biomarkers and gene expression signatures for prognostics or therapy are inadequate. Thus, there is a significant need to identify more factors associated with prognosis, to better personalize the choice of management strategies [8].

Protein tyrosine phosphatase receptor-type O (PTPRO), a type III member of the receptor-type PTP family, has come to the front as a tumor suppressor in multiple cancers [12,13]. Its encoding gene is frequently methylated and suppressed in breast cancer as well as in many other cancers [14-19]. Recent evidence shows that PTPRO takes part in estrogen action and has potential in enhancing tamoxifen sensitivity [16]. Furthermore, PTPRO regulates HER2 signaling and is involved in mammary morphogenesis; and lower PTPRO expression levels correlate with poor clinical outcome in HER2-positive patients [20]. These findings add new clues into understanding the role of PTPRO in breast cancer and signify its clinical prognostic value by indicating a new target for therapy in breast cancer. Nevertheless, to our knowledge no studies have investigated the prognostic value *per se* of PTPRO gene promoter methylation in patients with primary breast cancer. Therefore, the specific objective in this study was to better characterize the clinical relevance of PTPRO methylation in breast cancer. To do this, we determined the methylation status of the PTPRO gene promoter in 221 Chinese women with sporadic breast cancer and investigated whether PTPRO methylation was associated with clinicopathologic parameters and clinical outcome. In order to access its clinical application potential as a biomarker in peripheral blood, we further examined PTPRO in 24 matched plasma samples from breast cancer patients and 10 plasma samples from a normal control cohort.

## Materials and methods

### Human subjects and tissue specimens

A total of 221 breast tissue specimens were harvested and preserved from patients between June 1998 and December 2010 at the Affiliated Cancer Hospital of Shantou University Medical College. The median age of the patients was 51 years (range 21–89 years). Among the 221 cases, 123 women were premenopausal and 98 were postmenopausal. Histologically, all cases were invasive ductal carcinomas. At the time of operation, 41 cases (18.5%) were grade I tumors, 79 (35.7%) cases were grade II, and 83 cases (37.5%) were grade III. The pT stage of patients at initial diagnosis was stage 1 in 12 patients, 2 in 118, 3 in 22, and 4 in 18; in 5 cases the pT stage was not available. The pathological stage was assessed by surgical clinicians based on pathological reports and according to the 2003 TNM classification criteria by the International Union Against Cancer. A total of 74 cases (48.8%) were lymph node-negative, 41 cases (21.6%) were N1, 43 (10.0%) were N2, and 16 cases (19.6%) were N3. All patients, unless deceased, were followed up for at least 15 months and up to 124 months (mean 45.81 ± 17.91). All patients received conventional postoperative treatments, depending on the extent of the disease. The patients with ER+/PR + tumors were treated for 2–5 years with tamoxifen. The outcome was defined by the months of overall survival (OS) post-surgery. Preoperative peripheral blood (5 ml) from each patient was collected into an EDTA tube for the isolation of plasma. Control blood samples were obtained from 10 healthy volunteers. The use of human tissues in this study was approved by the Academic Committee of Shantou University Medical College. Patients who died of causes unrelated to the disease were not included in the study.

### Sample processing and genomic DNA bisulfite treatment

Paraffin blocks were cut to 8 μm-thick sections. In order to avoid cross contamination, a special procedure was employed in section cutting whereby new blades were used for each sample, and apparati, such as microtomes and tweezers, were carefully cleaned and disinfected between sample processing [21,22]. Two sections were collected into 1.5-ml microcentrifuge tubes. After xylene deparaffination and treatment with absolute ethanol, the sections were digested at 55°C overnight with proteinase K (0.1 mg/mL) in 200 mL of DNA extraction buffer. Bisulfite treatment was then performed with 20 μl of digestion supernatant, using an EZ DNA Methylation-Direct™ Kit (Zymo, Beijing) to convert unmethylated cytosine to uracil.

Paraffin blocks of PTPRO-methylated MCF-7 human cancer cell lines and PTPRO-unmethylated NE-2 immortalized normal cell lines were used as positive and negative controls, respectively, throughout the procedure (including FFPE block slicing, DNA extraction, bisulfite modification, and methylation-specific PCR).

Blood samples were centrifuged at 3000 × g, and plasma was carefully transferred into plain polypropylene tubes and stored at -70°C until further processing. DNA from plasma samples was extracted using a TIANamp Blood DNA Kit (TIANGEN, Beijing), following the blood and body fluid protocol as recommended by the manufacturer (8). The plasma samples (400 μl/column) were used for DNA extraction. A final elution volume of 50 μl was used. DNA (1 μg) from each sample was subjected to bisulfite modification through the use of an EZ DNA Methylation-Gold Kit (Zymo Research) following the manufacturer’s instructions.

### PTPRO gene promoter methylation analysis

Methylation-specific PCR (MSP) was performed with bisulfite-converted genomic DNA isolated from 221 FFPE specimens, using primers designed by Motiwala [17]: nonmethylation-specific primers hPTP-UF (5′- ATGTTTTTGGAGGATTTTGGGT-3′) and hPTP-UR (5′- ATACCCCATCACTACACAAACA-3′) and methylation-specific primers hPTP-MF (5′- CGTTTTTGGAGGATTTCGGGC-3′) and hPTP-MR (5′- AAAACACGACTACGCTAACG-3′) amplify 201- and 170-bp products respectively. Thermocycling conditions were modified according to Motiwala′s method and carried out in a 50-μl reaction containing ≈ 100 ng of bisulfite-converted DNA, PCR buffer, 10 pmol each of forward and reverse primers, 0.2 mM dNTPs, and 0.75 units of HotStarTaq® Plus DNA Polymerase (Qiagen, Valencia, CA). After an initial incubation at 94°C for 3 min, a touch-down PCR with 10 cycles of denaturation at 94°C for 30 sec, annealing at 55°C (Δ –0.4°C per cycle) for 1 min (Δ –0.04 sec per cycle), and extension at 72°C for 30 sec was performed, followed by an additional 35 cycles of denaturation for 30 sec at 94°C, annealing for 15 sec at 50.4°C, and extension for 30 sec at 72°C. The reactions ended with a 3 min final extension at 72°C, and then 10-μl aliquots of the PCR reaction were subjected to electrophoresis on a 2.5% agarose gel. DNA was visualized by ethidium bromide staining and captured by a BioRad Gel Doc™ XR imaging system.

### Statistical analyses

The association between methylation status and other molecular and clinicopathological parameters was calculated using contingency table methods and tested for significance using the chi-square test for categorical variables. Overall survival curves were calculated using the Kaplan-Meier method and the log-rank test was used to compare survival curves. Multivariate analysis was carried out using the Cox proportional hazard regression with a confidence interval of 95% to examine whether PTPRO methylation status was an independent prognostic factor for survival. All calculations were performed with SPSS 17.0 software (SPSS Inc., Chicago, IL, USA) for Windows. P-values less than 0.05 were considered to be statistically significant (two-sided).

## Results

### Prevalence of PTPRO methylation in sporadic primary breast cancer tissues

**Figure 1:**
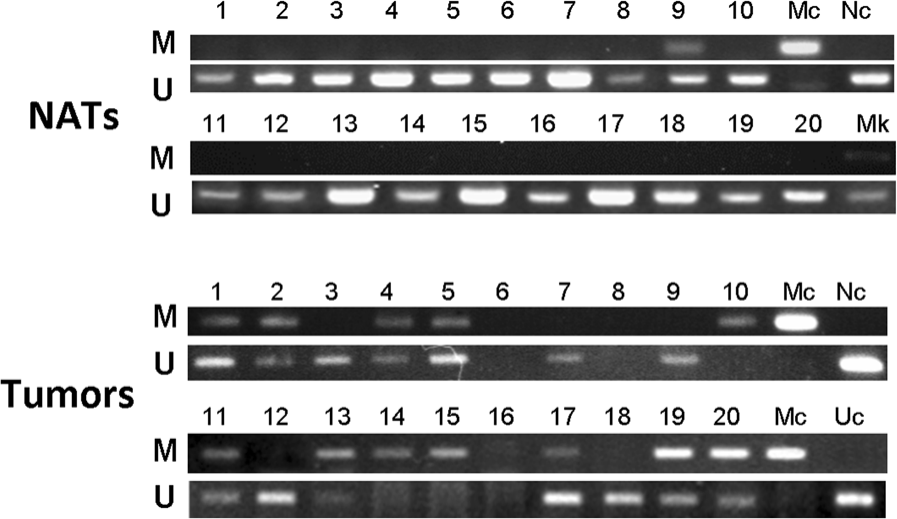
**Representative MSP assay for PTPRO gene methylation in FFPE specimens of normal adjacent tissues and tumors from breast cancer patients.** Numbers on top indicate sample number. NATs, Normal adjacent tissues; Mc, methylated control, paraffin blocks of MCF-7 human cancer cells; Uc, unmethylated control, paraffin blocks of NE-2 immortalized normal cells; M, methylated; U, unmethylated; Mk, DNA markers.

MSP analysis resulted in an identifiable product in 175 cases. Among these cases, methylation of the PTPRO promoter was detected in 130 (74.3%) of the 175 tumors. Among the tumors positive for promoter methylation, 35 (27%) were positive only for the methylated reaction and 95 (73%) were positive for both the unmethylated and methylated reactions. For the 20 adjacent normal tissues, only 1 (5%) displayed the methylation amplicon. A chi-square test suggested hypermethylation of PTPRO promoter occurs specifically and frequently in primary breast cancer (*P* < 0.000). Figure 1 shows a representative assay for PTPRO promoter methylation by methylation-specific PCR.

### Correlation of PTPRO methylation with clinicopathologic parameters

The clinical characteristics of the 175 breast cancer patients at the time of surgery are summarized in Table 1. Chi-square testing revealed a statistically significant association between the PTPRO promoter methylation and tumor grade (*P* = 0.028), whereas there was no significant correlation between PTPRO promoter methylation and age, pT, pN, stage, ER, PR, HER2 or menopausal status. Patients with lymph node metastasis (pN), younger age, premenopausal status, loss of ER and PR in tumors, larger tumor size (pT), and advanced stage showed a trend toward greater hypermethylation, but the trend was not significant (*P* = 0.051, 0.103, 0.123, 0.119, 0.118, 0.146, 0.250, respectively).

**Table 1:** Correlations of PTPRO methylation with different clinicopathological parameters

	**Total no.**	**PTPRO methylated**		**PTPRO unmethylated**		**χ**^**2**^	***P *-value**
		**NO**	**%**	**NO**	**%**		
All		175	130	74.3	45	25.7	
Age at diagnosis, years							
<45	56	46	82.1	10	17.9	2.662	0.103
> = 45	119	84	70.6	35	29.4		
pT							
0-1	12	7	58.3	5	41.7	3.885	0.146
2	118	93	78.8	25	21.2		
3-4	40	27	67.5	13	32.5		
NA	5	3		2			
pN							
0	74	51	68.9	23	31.1	7.75	0.051
1	41	27	65.9	14	34.1		
2	43	37	86.0	6	14.0		
3	16	14	87.5	2	12.5		
NA	3	2		1			
Clinical stage							
I + II	95	68	71.6	27	28.4	1.325	0.25
III	77	61	79.2	16	20.8		
NA	3	1		2			
Tumor grade							
G1 + G2	105	72	68.6	33	31.4	4.481	**0.028**
G3	67	56	83.6	11	16.4		
NA	3	2		1			
Estrogen receptor							
negative	53	43	81.1	10	18.9	2.432	0.119
positive	105	73	69.5	32	30.5		
NA	17	14		3			
Progesterone receptor							
negative	84	66	78.6	18	21.4	2.448	0.118
positive	74	50	67.6	24	32.4		
NA	17	14		3			
HER2							
negative	80	59	73.8	21	26.3	0.009	0.924
positive	78	57	73.1	21	26.9		
NA	17	14		3			
Menopausal status							
premenopausal	94	75	79.8	19	20.2	2.376	0.123
postmenopausal	79	55	69.6	24	30.4		
NA	2	0		2			

Bold values indicate significance.

### Prognostic value of breast cancer subtypes determined by PTPRO methylation status

**Figure 2:**
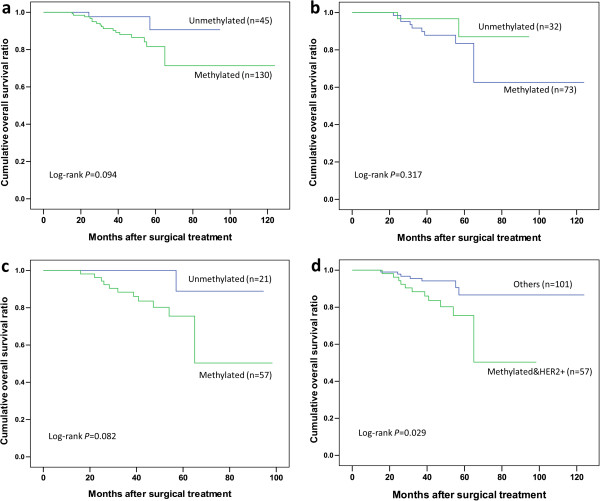
**Kaplan-Meier survival curves for overall and stratified subpopulations.** Kaplan-Meier survival curves for overall and stratified subpopulations in 175 patients according to the categories of PTPRO gene promoter methylation status (log-rank test analysis). **(a)** Overall survival (OS) for all patients, **(b)** OS for patients with ER-positive patients, **(c)** OS for patients with HER2-positive tumors, and **(d)** OS comparing patients with PTPRO-methylated/HER2+ tumors and remaining patients of the 175 cohorts.

Using a univariate Cox proportional hazard analysis, we examined clinicopathologic parameters, PTPRO methylation, and their association with overall survival end points. As summarized in Table 2, there were trends but no significant prognostic effects for PTPRO methylation in the 175 cohorts (HR, 3.273, CI 95%, 0.754-14.209; *P* = 0.113). Concerning the biological role of the PTPRO in the ER pathway and HER2 signaling, we stratified the entire patient cohort into subpopulations according to ER and HER2 status. In ER-positive patients, the unmethylated-PTPRO group did not show a trend for a more favorable outcome (n = 95; HR, 2.149; CI 95%, 0.463-9.983; *P* = 0.329). When tumor were HER2-positive, patients with methylated-PTPRO had a tendency for poor overall survival compared to those of unmethylated-PTPRO, but the differences were not significant (n = 78, HR, 5.112; CI 95%, 0.658-39.723; *P* = 0.119). Further analysis according to histological grade could not find additional significant difference (see Additional file 1: Figure S1). Kaplan-Meier curves for the above subpopulations according to PTPRO methylation are shown in Figure 2b, 2c and Additional file 1: Figure S1. These inconclusive results may be due to the limited sample size and restricted follow-up period. We then compared patients with combined methylated-PTPRO and HER2-positivity to the remaining patients in the 175 cohorts (unmethylated-PTPRO/HER2-positive patients and patients with HER2-negative tumors irrespective of the PTPRO methylation status). Significantly higher risk was observed for the methylated-PTPRO&HER2-positive group of patients (HR, 2.749, CI 95%, 1.065-7.097; P = 0.037). To confirm the significance of this finding, we performed multivariate analysis, treating methylated-PTPRO/HER2-positivity as a factor with tumor size, lymph node metastasis, histological grade and HER2 status for their impact on overall survival. After adjustment for these covariates, methylated-PTPRO/HER2-positivity was identified as an independent predictor for overall survival (HR, 3.663; CI 95%, 1.371-9.784; *P* = 0.010). Similarly, tumor size (pT) and lymph node metastasis (pN) also had an independent association with overall survival in this patient cohort. Kaplan-Meier survival curves showed that promoter methylation of the PTPRO gene plus HER2-positivity was associated with poor overall survival (*P* = 0.029; Figure 2d).

**Table 2:** Univariate and multivariate Cox proportional hazards model for overall survival in breast cancer patients (n = 175)

**Clinicopathological parameters**	**Univariate analysis**			**Multivariate analysis**			
	**Hazard ratio**	**CI 95%**	***P*-value**	**Hazard ratio**	**CI 95%**	***P-*value**	
Estrogen receptor							
positive (vs. negative)	0.737	0.285-1.906	0.530				
Progesterone receptor							
positive (vs. negative)	0.559	0.214-1.458	0.235				
HER2							
positive (vs. negative)	1.765	0.661-4.716	0.257				
Hormone receptor							
positive (vs. negative)	0.737	0.285-1.906	0.530				
Age							
> = 45 (vs. <45)	1.886	0.626-5.684	0.260				
Histological grade							
III (vs. II & I )	2.572	1.238-5.342	**0.011**				
pT							
4 & 3 (vs. 2 & 1)	1.724	0.996-2.985	0.052	1.77	1.005-3.118	**0.048**	
pN							
positive (vs. negative)	4.343	1.263-14.933	**0.020**	4.40	1.238-15.673	**0.022**	
Stage							
III (vs. II & I )	1.985	0.878-4.489	0.100				
PTPRO methylation							
methylated (vs. unmethylated)	3.273	0.754-14.209	0.113				
PTPRO methylation & HER2 status							
Methylated/HER2+ (vs. others)	2.749	1.065-7.097	**0.037**	3.663	1.371-9.784	**0.010**	

3 cases were censored cases before the earliest event in a stratum.

Clinical tumor size and nodal status according to TNM staging system.

Bold values indicate significance. *CI*, Confidence interval; HER2, human epidermal growth factor receptor 2.

### PTPRO methylation as a peripheral blood biomarker for breast cancer

**Figure 3:**
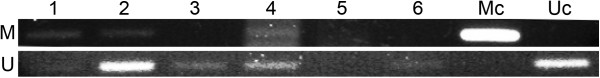
**Representative MSP assay for PTPRO gene methylation detected in matched plasma samples.** Numbers on top indicate sample number. Mc, methylated control; Uc, unmethylated control; M, methylated; U, unmethylated.

We further explored the feasibility of detecting PTPRO methylation in the plasma of matched peripheral blood samples from breast cancer patients (Figure 3). Among 24 matched plasma samples, PTPRO was aberrantly methylated in 11 (45.8%) (Table 3), and detected only in plasma from patients whose corresponding primary tumors were also methylated. Additionally, no methylation of PTPRO was observed in any normal control plasma samples from 10 healthy individuals.

**Table 3:** Methylation patterns of the PTPRO genes in primary tumors, plasma samples from 24 breast cancer patients with Clinicopathological parameters

**Patient NO.**	**Tumor**	**Plasma**	**HER2**	**ER**	**PR**	**Age**	**Grade**	**pT**	**pN**	**pM**
1	●	●	+	-	-	52	2	2	1	0
2	●	●	+	-	-	49	2	2	0	0
3	●	●	+	-	-	41	2	3	1	0
4	●	●	-	-	-	41	3	2	0	0
5	●	●	-	+	+	89	3	4	x	0
6	●	●	-	+	-	58	3	4	2	0
7	●	●	-	+	-	57	2	4	2	0
8	●	●	-	+	-	57	1	2	0	0
9	●	●	-	+	-	54	2	2	0	0
10	●	●	-	+	+	30	3	3	3	0
11	●	●	-	-	-	48	1	4	2	0
12	●	○	+	+	+	42	3	4	2	0
13	●	○	+	-	-	52	2	2	0	0
14	●	○	+	+	+	45	2	4	2	0
15	●	○	-	+	+	57	2	2	1	0
16	●	○	-	+	-	62	2	2	2	0
17	●	○	-	+	+	46	2	4	3	0
18	●	○	-	+	+	60	3	2	2	0
19	○	○	+	+	-	42	1	2	1	0
20	○	○	+	-	-	61	3	3	0	0
21	○	○	-	+	-	36	2	3	3	0
22	○	○	-	+	+	50	1	1	0	0
23	○	○	-	+	-	54	3	3	0	0
24	○	○	-	+	-	54	2	1	0	0
Methylation ratio (%)	18/24 (75.0)	11/24 (45.8)								

●, Methylated; ○, unmethylated.

## Discussion

A previous study examined 21 breast cancer specimens and found 17 cases (81%) that had dense hypermethylation in the CpG island of the PTPRO gene, whereas the adjacent normal tissue remained unmethylated [16]. We extend these studies, using a much larger sample size, to show a similar 74.3% (130/175) incidence of PTPRO methylation in breast cancer. Our larger sample size enables us to conclude that the PTPRO methylation is a common event in primary breast cancer.

Similar to our previous data showing that PTPRO methylation correlates with advanced stage in esophageal squamous cell carcinoma [15], our clinicopathologic correlation analysis, showing that PTPRO promoter methylation correlates with tumor histological grade (*P* = 0.028) suggests that the PTPRO silencing influences breast cancer differentiation and more directly correlates with aggressive biological behaviour of tumor. Thus, the precise downstream effects of PTPRO methylation in the carcinogenesis and progression of breast cancer deserves further exploration.

To our knowledge, this is the first report that investigated the prognostic value per se of PTPRO gene promoter methylation in patients with primary breast cancer [23-26]. Primary survival analysis of the entire cohort reveals no prognostic value for PTPRO methylation in breast cancer patients in general. In different stratified cohort populations, ER-positive patients with unmethylated PTPRO show statistically insignificant trend toward more favorable outcome compared to those with methylated PTPRO. This does not support prior in vitro studies showing PTPRO enhances tamoxifen sensitivity [16]. If this relationship could be confirmed, ER-positive patients could be stratified into more precise subpopulations to decide whether the patients need a regimen containing tamoxifen.

In contrast to the ER-positive group, we first verified that patients with methylated-PTPRO in the HER2-positive group have a significantly higher risk for mortality compared to the remaining patients in the cohort. Multivariate analysis identified methylated PTPRO/HER2-positivity as an independent predictor for overall survival. Our finding of PTPRO promoter methylation, which would suppress the PTPRO gene expression, as a cause of poor survival also explains prior data mining studies showing low PTPRO expression correlates with poor clinical prognosis in HER2-positive patients [16,25]. These observations have major implications for breast cancer patients with amplified/overexpressed HER2 and unmethylated PTPRO, targeted drugs therapies (e.g., Trastuzumab and Lapatinib) may prove ineffective by failing to provide extensive survival benefits due to active PTPRO. It would either cut down the costs needed to treat patients or reduce the risk of side effects caused by target drugs. To clearly address this question, further studies using patients of the cohort who receive Trastuzumab therapy are required.

Analysis of matched plasma samples shows that methylated PTPRO can be utilized as a peripheral tumor biomarker for noninvasive diagnosis and disease monitoring. However, the sensitivity of detection in the plasma fraction appears to be less than that in tumor tissues [11/18(61.1%)]. On the other hand, when compared with detection of expression levels in primary tumor tissue by RT-PCR or immunohistochemistry, the methylation approach outlined here has the advantages of ease of implementation for either screening of breast cancer in early stages, or monitoring resistance or relapse [27].

Further validation in a larger series of breast cancer patients will advance molecular subtyping and enhance our understanding of the clinical behavior of these tumors, as well as provide targets for better therapy [6]. Due to its clinical prognostic value, PTPRO gene promoter methylation represents a new diagnostic tool to be added to other clinicopathological and molecular variables for predicting patients outcome or optimizing individualized therapy [28-31].

## Conclusions

PTPRO methylation is a common event in primary breast cancer and can be reliably detected in peripheral blood samples. PTPRO methylation is associated with unfavorable survival in patients with HER2-positive breast tumors, and therefore can serve as a potential prognostic factor for breast cancer.

## Abbreviations

PTPRO: Protein tyrosine phosphatase receptor-type O; HER2: Human epidermal growth factor receptor-2; ER: Estrogen receptor; PR: Progesterone receptor; RT-PCR: Reverse transcription polymerase chain reaction; MSP: Methylation-specific PCR.

## Competing interests

The authors declare that they have no competing interest.

## Authors’ contributions

HYT carried out experiments, analyzed the data, and participated in the experiments design and manuscript writing. CK and YPC contributed reagents, materials, and analysis tools. FFL, ZL and XXZ collected data, and participated in experiment performance. ZTL and XFZ participated in the sample preparation, data collection and interpretation of the data. HZ conceived the study, designed experiments and wrote the manuscript. All authors read and approved the final version of the manuscript.

## Supplementary Material

Additional file 1: Figure S1 Kaplan-Meier survival curves for stratified subpopulations according to histological grade. Kaplan-Meier survival curves for overall and stratified subpopulations in 175 patients according to the categories of PTPRO gene promoter methylation status (log-rank test analysis). (e) Overall survival (OS) for patients with Grades 1 and 2 tumors, (f) OS for patients with Grade 3 tumors. Click here for file
