# Supplementary figures and images for: PTPRO promoter methylation is predictive of poorer outcome for HER2-positive breast cancer: indication for personalized therapy
Source: J Transl Med. 2013 Oct 3;11:245. doi: 10.1186/1479-5876-11-245 (PMC3852714; doi:10.1186/1479-5876-11-245)

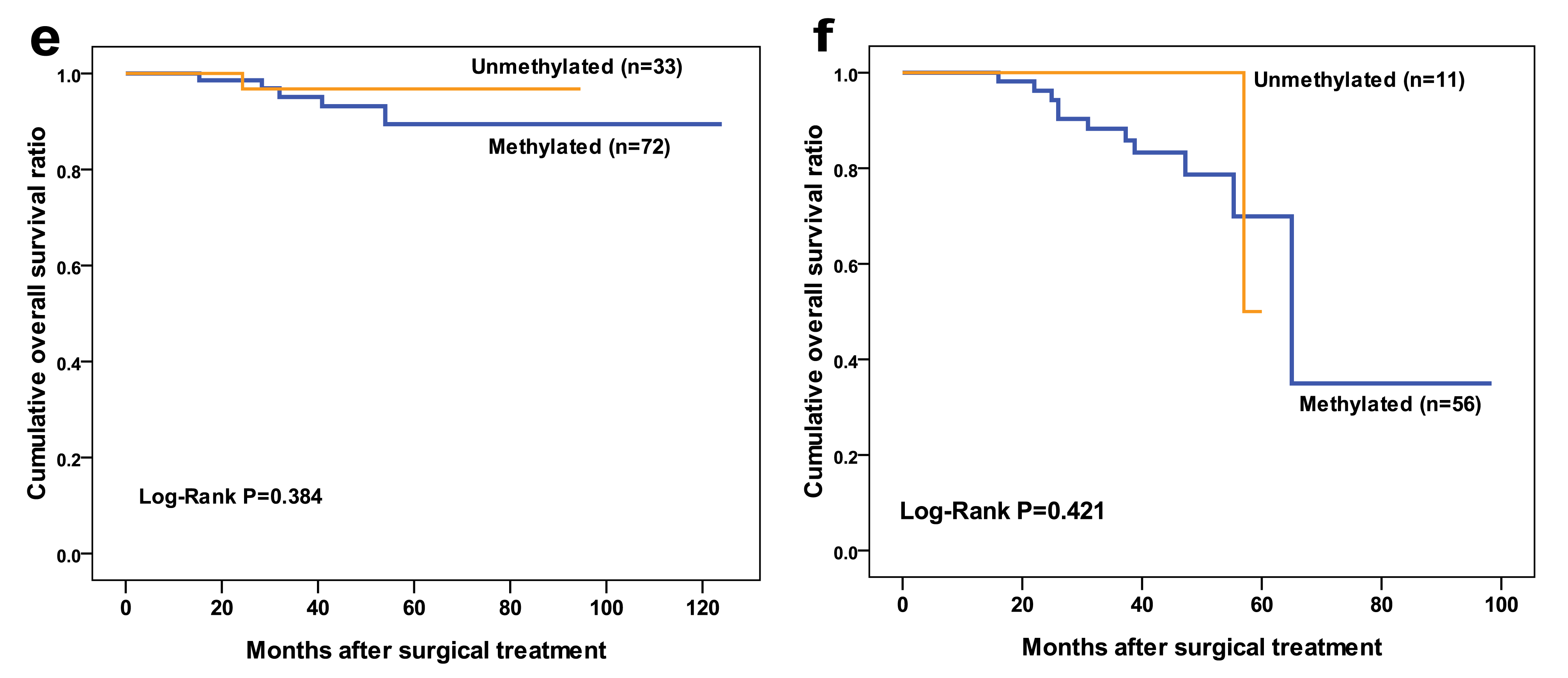

Supplement: Additional file 1: Figure S1 — Kaplan-Meier survival curves for stratified subpopulations according to histological grade. Kaplan-Meier survival curves for overall and stratified subpopulations in 175 patients according to the categories of PTPRO gene promoter methylation status (log-rank test analysis). (e) Overall survival (OS) for patients with Grades 1 and 2 tumors, (f) OS for patients with Grade 3 tumors. [file 1479-5876-11-245-S1.tiff]
